# Urinary Deposits, Their Diagnosis, Pathology, and Therapeutical Indications

**Published:** 1845-04

**Authors:** 


### URINARY DEPOSITS, THEIR DIAGNOSIS, PATHOLOGY, AND THERAPEUTICAL INDICATIONS. By *Golding Bird,* A.M. M.D. Assistant Physician to, and Lecturer on Materia Medica at Guy's Hospital. Royal 12mo. pp. 323. Churchill, London, 1844.

WE have for some time past felt the want of a book on urinary diseases, which should give to the practical physician a general view of the recently advanced chemico-pathological doctrines of the Giessen school, in so far at least as they relate to alterations in the conditions of the renal secretion; and point out to him all the available and useful practical hints which are derivable from the present greatly improved state of chemical physiology.

The followers of Liebig have boldly declared that the hypotheses of their master are capable of great and valuable applications to the improvement of practical medicine; and we have been long desirous of seeing the whole subject carefully investigated by a practical man, and one who, though acquainted with, is notwithstanding unbiassed by, the fashionable chemical doctrines of the day.

Dr. Golding Bird has been for some years favorably known to the profession as an active and highly intelligent physician, who has not confined his studies to what is commonly termed the practical part of his profession, but has, in addition, diligently and very successfully studied the physical and chemical sciences. That the knowledge which he has thus obtained has been usefully applied to the purposes of practical medicine, the work, whose title heads this article, bears ample evidence. It is on a subject peculiarly well adapted for his attainments; for there is, we believe, no department of medicine to which physical and chemical acquirements can be so usefully applied as that which relates to urinary sediments; and we regret to be obliged to add that there is no class of medical subjects on which physicians in general have less knowledge, both theoretical and practical, than that of urinary diseases.

In the year 1843, Dr. Bird delivered, to the pupils of Guy's Hospital, a short course of six lectures on the diagnosis and pathology of urinary sediments, which were published in the *London Medical Gazette.* These lectures form the basis of the present volume; though Dr. Bird tells us that the subject is greatly extended and nearly re-written.

Dr. Bird's work does not supply all the information relating to abnormal conditions of the urinary secretion, which recent investigations have supplied us; for it professes to limit its range to deposits in the urine. But we are bound to confess that, as far as it goes, it furnishes much useful matter of the kind greatly needed.

In addition to some introductory remarks on the clinical examination of urine, and an appendix containing some tables and references, Dr. Bird's work consists of eleven chapters on the following subjects: 1, Physiological Origin and Physical Properties of the Urine; 2, Chemical Physiology of the Urine; 3, Chemical Pathology of Uric Acid and its Combinations; 4, Chemical Pathology of Uric Oxide; 5, Chemical Pathology of Purpurine; 6, Chemical Pathology of Cystine; 7, Chemical

Pathology of Oxalate of Lime (Oxaluria); 8, Chemical Pathology of the Earthy Salts; 9, Deposits of Black or Blue Colouring Matters; 10, General Pathology of Non-Crystalline Organic and Organised Deposits; and 11, Therapeutical Employment of Remedies influencing the Kidneys.

We propose to notice more or less in detail each of these chapters in the order in which they stand in the original work.

CHAP. 1. *Physiological Origin and Physical Properties of Urine.*—Dr. Bird commences this chapter with some judicious observations on the indications afforded by the renal secretion; and cautions the practitioner against regarding every abnormal condition of the urine as being in itself a disease, since it is in fact merely a symptom,—an indication of some particular phase of morbid action.

The kidneys perform three important functions: they carry off any excess of fluid which may enter the circulation; they remove any crude or indigested elements of the food, as well as the results of imperfect or unhealthy assimilation; and, lastly, they serve to throw off certain highly nitrogenised combinations formed by the re-arrangement of the atoms of the old tissues.

"It is therefore necessary to recognise three distinct varieties of the urinary secretion in every case under investigation: Firstly, that passed some little time after drinking freely of fluids, generally pale, and of low specific gravity, (1.003—1.009) *urina potus*. Secondly, that secreted after the digestion of a full meal, varying much in physical characters and of considerable density, (1.020—1.028 or even 1.030,) *urina chyli vel cibi*. Thirdly, that secreted from the blood independently of the immediate stimulus of food and drink, as that passed after a night's rest, *urina sanguinis;* this is usually of average density, (1.015—1.025,) and presents in perfection the essential characters of urine." 5.

After giving a slight notice of Dr. Prout's ideas respecting primary and secondary assimilation, and of his opinion that uric acid and urate of ammonia are derived from the metamorphosis of the albuminous tissues, while urea and some saccharine principle or its close ally lactic acid, are produced by the gelatinous tissues, Dr. Bird sketches briefly, but clearly, the views of Liebig, and Mulder on this subject. Liebig, he says:—

"Has assumed that the ultimate composition of animal flesh, as a muscle, and of blood, can be expressed by the same formula, and are consequently chemically identical. When, therefore, animal fibre is taken into the stomach, it undergoes a kind of imperfect solution, and reaches the circulation, possessing nearly the same chemical composition as the blood with which it becomes mixed. It then undergoes certain changes in the lungs, assuming probably a more highly vitalised condition connected essentially with the conversion of its albumen into self-coagulating fibrin; bodies, however different in their physical and molecular arrangement, identical in composition. Reaching in their course the nutrient capillaries, the elements of the food are deposited in the substance of a tissue, as a muscle, whose waste they thus supply. Ere these new molecules can be deposited, room must be made for them by the removal of old matter, and then the following beautiful results of vital chemistry are supposed to come into play. The exhausted atoms of the muscle cannot be removed as fibres, but their elements must be re-arranged, so as to enter the circulation and be carried to other organs. They therefore undergo metamorphosis; water and oxygen are conveyed to the muscle, the former in the fluid of the blood, the latter in the red particles, and the result is the re-arrangement of elements, which, whilst it enables the old tissue to be removed with facility, furnishes the pabulum for other and important secretions.

"The late researches of Professor Mulder of Utrecht on the combination of protein with oxygen, have thrown much light on a very obscure part of the act of metamorphosis of tissues, and which constituted the least tenable part of Liebig's hypothesis: he having, as already stated, assumed that oxygen is conveyed to the capillaries in the arterial blood-corpuscles, combined with iron, as sesqui-oxide—which giving up part of its oxygen, reaches the venous blood as protoxide. This idea can be only regarded as an ingenious assumption, for which no proof is offered by its talented author. All the elements of our food capable of being organised into albuminous tissues, consist of protein ($C_{48}\ N_6\ H_{36}\ O_{14}$) combined with varying proportions of sulphur and phosphorus. Professor Mulder has discovered two oxides of protein, a binoxide and tritoxide, both of which are formed in the animal economy, and constitute, when combined with fatter matter, the buffy coat of inflamed blood. He believes that the protein of the food reaches the right side of the heart, circulates through the lungs, and combines with oxygen, forming oxy-protein (binoxide, tritoxide, or both); this reaches the nutrient capillaries, and all or part is decomposed; the oxygen being employed for the disorganisation of worn-out tissue, the protein thus de-oxidised being deposited to supply its place. If more protein is set free than is wanted for the growth of tissue, it passes unchanged into the veins, to be again oxidised in the lungs. The tritoxide of protein being soluble in water, is better enabled to traverse the minutest capillaries than if it existed merely diffused through the fluid containing it." 9.

The greater part of the remaining portion of this chapter is taken up with details respecting the physical properties (density, colour, and consistence) of the urine. He notices the proposed application of the polariscope to the detection of sugar in the urine; and observes that, there are many practical difficulties in the application of the polarising power of urine to the detection of sugar, which will probably ever prevent its being generally employed. In this opinion we quite concur.

CHAP. II. *Chemical Physiology of the Urine.*—After making some general statements respecting the composition of the urine, Dr. Bird proceeds to examine individually each of the essential and important constituents of this secretion, pointing out its principal physical and chemical characters and its physiological and pathological origin.

In speaking of *urea*, he observes that the physiological origin of this substance is owing to the destructive assimilation of the tissues of the body.

"That urea is one of the products of this important process, and that it constitutes the mode in which the greatest portion of the nitrogenised elements are secreted, is unquestionable. In man and warm-blooded, carnivorous and omnivorous mammalia, its quantity far exceeds that of uric acid; whilst, in carnivorous birds, serpents, and insects, the latter substance predominates, and often quite replaces the urea. Dr. Prout is inclined to believe that the urea is the peculiar product of the metamorphosis of gelatinous, and uric acid of albuminous, structures. Liebig, on the other hand, considers that uric acid is the immediate product of the change in all nitrogenised tissues, and that urea is the secondary product, arising from the action of oxygen and water in the uric acid. The fact that in sea-birds and many insects the uric acid remains in the state of urate of ammonia, and does not become converted into urea, notwithstanding all the conditions necessary on Liebig's views for this change exist, must cause this hypothesis to be received with great caution." 34.

The influence of food in modifying the quantity of urea in the urine has been clearly shown by the experiments of Dr. Lehmann made on himself. The mean number of grains of urea obtained from the urine in 24 hours is thus expressed :—

| Diet. | Animal. | Vegetable. | Mixed. | Non-nitrogenised. |
|---|---|---|---|---|
| Urea in the urine of 24 hours | 819.2 | 346.5 | 500.5 | 237.1 |

"No one can avoid observing the great disproportion existing between the quantity of urea existing in Lehmann's urine, and that generally met with; the quantity secreted whilst confined to a strictly non-azotised diet, nearly equalling the normal proportion." 35.

These observations are exceedingly valuable, in a practical point of view. They suggest the important aid to be derived from attention to diet, in the treatment of the disease called by Dr. Willis *Azoturia;* but of which Dr. Bird takes no notice.

Of the mode in which *uric acid* exists in healthy urine, Dr. Bird takes the following view :

"*Uric acid, at the moment of separation from the blood, meets the double phosphate of soda and ammonia, derived from the food, and forms urate of ammonia evolving phosphoric acid, which thus produces the natural acid reaction of urine. If the whole bulk of the urine be to the urate of ammonia formed, not less than about 2700 to* 1, *the secretion will, at the ordinary temperature of the air, remain clear, but if the bulk of fluid be less, an amorphous deposit of the urate will occur. On the other hand, if an excess of uric acid be separated by the kidneys, it will act on the phosphate of soda of the double salt, and hence, on cooling, the urine will deposit a crystalline sediment of uric acid sand, very probably mixed with amorphous urate of ammonia, the latter usually forming a layer above the crystals, which always sink to the bottom of the vessel.*" 42.

Liebig ascribes the origin of uric acid to the chemical action of oxygen of the arterial blood on the protein tissues. The following equation shews the products of this reaction.

| Protein. | | Oxygen. | | Uric Acid. | | Carbonic Acid. | | Water. |
|---|---|---|---|---|---|---|---|---|
| $C^{48} N^{6} H^{36} O^{14}$ | $+$ | $O^{91}$ | $=$ | $1\frac{1}{7} (C^{10} N^{4} H^{4} O^{6})$ | $+$ | $33 (CO^{2})$ | $+$ | $30 (HO.)$ |

Dr. Bird rejects, and very properly as we conceive, Liebig's hypothesis, and he observes that—

"If these views be correct, it will follow that, other things being equal, the proportion of uric acid in the urine will increase in the urine of a man who takes food rich in carbon, and decrease if he confines himself to a nitrogenised diet, thus becoming a carnivorous animal. Further, the proportion of uric acid will decrease and urea increase, with the perfection of respiration and abundance of blood-discs, the reputed carriers of oxygen.

"It appears to me, however, that these views, ingenious and full of interest as they are, are not supported by any experience hitherto recorded, in fact, are, in many cases, totally opposed by it. The experiments of Lehmann, already alluded to, performed upon himself, demonstrate that vegetable diet and one quite free from nitrogen decreases, and an animal diet increases the quantity of uric acid; the urea also increases in the same manner. The following table presents the results of Lehmann on himself.

| DIET. | Quantity excreted in 24 hours of | | Proportion of Uric Acid to Urea. |
|---|---|---|---|
| | URIC ACID. | UREA. | |
| Exclusively animal .. .. | 22.64 grs. | 819.2 grs. | 1 : 36.1 |
| Mixed animal and vegetable | 18.17 .. | 500.5 | 1 : 27.5 |
| Exclusively vegetable.. .. | 15.7 .. | 346.5 | 1 : 22. |
| Food free from nitrogen .. | 11.24 .. | 237.1 | 1 : 21. |

*Hippuric acid* has only recently been satisfactorily shewn to constitute an ingredient of healthy urine. Its physiological origin is believed to be some of the non-azotised constituents of the food; and in proof of this Dr. Bird mentions a curious fact communicated to him by Liebig. A girl, labouring under what appears to have been some form of hysteria, refused all food, excepting apples, of which she devoured an enormous quantity. On examining her urine, it was found to be alkaline, and contained a large quantity of hippuric, but no uric acid, like the urine of a horse or cow.

By the use of either benzoic or cinnamic acid the quantity of hippuric acid is greatly augmented; a fact for which we are indebted to Mr. Alexander Ure. Hippuric acid contains the elements of urea *plus* those of lactic acid or of sugar or starch; a fact first explained by Dr. Garrod.

Dr. Bird takes no notice of any connexion between an abnormal quantity of hippuric acid in the urine and other symptoms of disease. We have reason to suspect, however, that some forms of dyspepsia are associated with an excess of hippuric acid.

*Butyric acid* has been found in diabetic urine. It is a well-known constituent of butter; and recently Pelouze and Gèlis have shown that it may be formed from sugar by the aid of a piece of casein or glutine to act as a ferment. It is obvious, therefore, that there are two possible sources of the butyric acid found in the urine, namely, butter taken as food, and a modified assimilation of sugar. Curiously enough, Dr. Bird only refers to the latter origin of it; totally omitting to suggest the more natural and obvious source of it, namely, the butter employed as food.

We have a particular reason for believing the probably dietetical origin of this acid. In some experiments which we have made on diabetic urine we succeeded in obtaining sugar from the urine, when, to the best of our belief, the patient's food contained neither sugar nor any substance known to contain or yield it; when, in fact, the diet was exclusively animal without milk.

In reflecting on this, we were led to suspect the possible conversion of glycerine into sugar. Now our readers are probably aware that butter, as well as some other animal fats, are composed of fatty acids combined with glycerine; and the fact of the existence of one, at least, of the fatty acids (viz. butyric acid) in the urine of diabetic patients lends some support to our hypothesis.

The existence of *Lactic Acid and Lactate of Ammonia* in the urine, as at one time admitted, has been recently denied by Liebig. It would appear that the substance which has been mistaken for these bodies is weakly basic, and contains a very large quantity of nitrogen.

But very little is known of the pigments of the urine. The yellow colouring matter is perhaps *hæmaphaein,* which is also found in the blood. The red pigment of the urine is *purpurine* (called *uro-erethrine* by Simon), which is sometimes confounded with murexid (purpurate of ammonia.)

The *phosphates* of the urine are derived either from the food or from the action of oxygen on those structures into which phosphorus enters as an essential constituent. The *sulphates* have a similar origin.

Dr. Bird concludes this chapter with a tabular view of urinary deposits.

" *Class* 1.—Deposits composed essentially of ingredients formed directly or indirectly from the metamorphosis of tissues, or from the organic elements of food.

Uric acid and urates.
Uric oxide.
Oxalate of lime.
Cystine.

" *Class* 2.—Deposits composed of ingredients of inorganic origin; including—

Phosphate of lime.
Ammonio-phosphate of magnesia.
Carbonate of lime.
Silicic acid.

" *Class* 3.—Highly coloured deposits (black or blue) of doubtful origin.

Cyanourine.

Melanourine.

Indigo.

Prussian blue.

" *Class* 4.—Deposits consisting of non-crystalline organic products; including—

A. *Organised.*
Blood.
Pus.
Mucus.
Organic globules.
Epithelium.

B. *Non-organised.*
Milk.
Fatty matter.

C. *Possessing independent vitality.*
Spermatozoa.
Torulæ.
Vibriones."

A worse and more unphilosophical classification we have never met with: the two first classes are founded on the *origin* of the deposits—the third class on their *colour*—and the fourth on their *external form!* Passing over the difficulty, in many cases, of determining the organic or inorganic origin of a deposit, it is obvious that Dr. Bird's two first classes include the deposits arranged under the two latter ones! Are not the blue and black pigments of the urine and the blood formed from the metamorphosis of tissues or from the organic elements of food?" If so, they are clearly referable to the first class. Are not cyanourine, indigo (as found in the urine), and melono urine "deposits consisting of non-crystalline organic products," and, therefore, referable to the fourth class? Moreover, in the subdivisions of the fourth class, Dr. Bird again evinces a complete ignorance of the principles of classification; for it is evident that, as he takes the presence or absence of organization as the basis of his subdivision, there can be but two orders; and it is obvious that all the deposits placed in his third subdivision are really referable to the first, seeing that all of them are organised.

Dr. Bird commences his work with some introductory remarks on the clinical examination of urine. As these are most concisely and neatly stated, and have, moreover, considerable practical utility, we shall offer no apology for transferring them to our pages; and the present seems to be the most appropriate place for introducing them. We may premise that the urine examined should be an average specimen of that passed in the preceding twenty-four hours, or at least that resulting from the first act of emission after a night's rest.

"*A.—Urine without any visible deposit.*

"A piece of litmus paper should be immersed in the urine, which if acid will change the blue colour of the paper to red. Should no change occur, a piece of reddened litmus paper must be dipped in, and if the secretion be alcaline, its blue colour will be restored; but if no change occur, the urine is neutral.

"Some of the urine should then be gently heated in a polished metallic spoon over a candle, or what is preferable, in a test-tube over a spirit-lamp, and if a white deposit occurs, albumen or earthy phosphates are present; the former, if a drop of nitric acid does not re-dissolve the deposit, the latter if it does.

"If the urine be very highly coloured, and undergoes no change by boiling, the colouring matters of bile, BLOOD, or purpurine are present. To determine which, pour a thin layer of urine on the back of a white plate, and allow a few drops of nitric acid to fall in the centre; an immediate and rapidly ending play of colours, from green to red, will occur if bile, but no such change if purpurine alone exists. Should the highly-coloured urine alter in colour or transparency by heat, the presence of blood must be suspected.

"If the addition of nitric acid to deep red urine, unaffected by heat, produces a brown deposit, an excess of uric acid exists. If the urine be pale, immerse the gravimeter, and if the specific gravity be below 1.012, an excess of water exists in the urine, but if above 1.025, the presence of sugar, or excess of urea is indicated. To determine which, place a few drops in a watch-glass, add an equal quantity of nitric acid, and allow the glass to float on some cold water; crystallisation of nitrate of urea will occur in two or three minutes, if the latter exists in excess. Should this change not occur, the urine must be examined specially for sugar, which, it must be remembered, may exist in small quantities, without raising the specific gravity of the fluid.

"Should the urine be alcaline, add a drop of nitric acid; if a white deposit occurs, albumen is present; if brisk effervescence follows the addition of the acid, the urea has been converted into carbonate of ammonia.

"*B.—Urine depositing a visible sediment.*

"If the deposit is flocculent, easily diffused on agitation, and scanty, not disappearing on the addition of nitric acid, it is chiefly made up of healthy mucus, epithelium, or in women, an admixture of leucorrhœal discharge.

"If the deposit is ropy and apparently viscid, add a drop of nitric acid; if it wholly or partly dissolves, it is composed of phosphates, if but slightly affected, of mucus. If the deposit falls like a creamy layer to the bottom of the vessel, the supernatant urine being coagulable by heat, it consists of pus.

"If the deposit is white, it consists of urate of ammonia, phosphates, or cystine; the first disappears on heating the urine, the second on the addition of a drop of diluted nitric acid, whilst the third dissolves in ammonia, and the urine generally evolves an odour of sweet-briar.

"If the deposit be coloured, it consists of red particles of blood, uric acid, or urate of ammonia, stained with purpurine. If the first, the urine becomes opake by heat; if the second, the deposit is in visible crystals; if the third, the deposit is amorphous, and dissolves on heating the fluid.

"Oxalate of lime is often present diffused through urine, without forming a visible deposit; if this be suspected, a drop of the urine examined microscopically will detect the characteristic crystals.

"Much of the little time required for the investigation thus sketched out, may be saved by remembering the following facts.

"If the deposit be white, and the urine acid, it in the great majority of cases consists of urate of ammonia; but should it not disappear by heat, it is phosphatic.

"If a deposit be of any colour inclining to yellow, drab, pink, or red, it is almost sure to be urate of ammonia, unless visibly crystalline, in which case it consists of uric acid.

"The only apparatus and re-agents required for these investigations at the bed-side are—

"A gravimeter, made small enough to float in an ounce of fluid.

"Red and blue litmus paper.

"A test-tube and watch-glass.

"Nitric acid.

"All these are readily arranged in a little case, and can thus be always at the convenience of the practitioner. For the microscopic examination of the urine, a vertical instrument on a firm tripod stand, and large ring-stage, provided with a good half-inch achromatic object-glass, is alone required.

"The following table briefly points out the best mode for the analytical examination of saline deposits, either by chemical tests or the microscope. The latter mode of investigation is infinitely preferable to all others, both for accuracy and economy of time, but is of course not readily available in the sick-room.

*A.—A Table for discovering the nature of saline deposits by chemical reagents.*

| | | |
|---|---|---|
| 1. Deposit, white | - - - - - - | 2. |
| ——— coloured | - - - - - | 5. |
| 2. ——— dissolves by heat | - - - | Urate of ammonia. |
| ——— insoluble by heat | - - - | 3. |
| 3. ——— soluble in liquor ammoniæ | - | Cystine. |
| ——— insoluble in | - - - | 4. |
| 4. ——— soluble in acetic acid | - - | Earthy phosphates. |
| ——— insoluble | - - - - | Oxalate of lime. |
| 5. ——— visibly crystalline | - - - | Uric acid. |
| ——— amorphous | - - - - | 6. |
| 6. ——— readily soluble by heat | - - | Urates. |
| ——— slowly dissolves by heat | - - | ——— stained by purpurine. |

*B.—Table for determining the nature of saline deposits by the microscope.*

| | | |
|---|---|---|
| 1. | Deposit, white - - - - - - | 2. |
| | ——— coloured - - - - - | 5. |
| 2. | ——— an amorphous powder - - | Insoluble by heat—Phosphate of lime. Soluble by heat—Urate of ammonia. |
| | ——— in defined crystals - - | 3. |
| 3. | ——— in prismatic crystals - - | Triple phosphate. |
| | ——— in octohedral or tubular crystals | 4. |
| 4. | ——— in octohedra - - - | Oxalate of lime. |
| | ——— in simple or compound tables - | Cystine. |
| 5. | ——— in transparent crystals - - | Uric acid. |
| | ——— amorphous, or in spherical masses | Urates of ammonia or soda." |

On these directions we have to observe that they contain one very remarkable (typographical ?) error. Dr. Bird states that, "if the urine be very highly coloured, and undergoes no change by boiling, the colouring matters of bile, *blood*, or purpurine are present." The statement is correct as regards bile and purpurine, but not with respect to blood; for it is well known, as Dr. Bird himself subsequently states, that bloody urine alters both in colour and transparency by heat. Moreover, the headings of these two tables are incorrect; for Dr. Bird cannot mean to assert that either *Uric acid* or *Cystine* are "saline" substances.

CHAP. III.—*Chemical Pathology of Uric Acid and its Combinations.*—In this chapter Dr. Bird examines deposits of Uric Acid, Urate of Ammonia, and Urate of Soda.

The first of these, viz. *Uric Acid*, invariably occurs in a crystalline form, though under some considerable variety of shapes, all referable, however, to modifications of the rhombic prism, which may be assumed to be the normal crystalline form of this acid. The crystals are never colourless, but always present some tint of yellow or red. They do not dissolve when heated in the urine, but are soluble in liquor potassæ as well as in nitric acid; and the nitric solution yields, by evaporation, a pink residue constituting the *murexid* of Liebig, the *purpurate of ammonia* of Prout.

*Urate of Ammonia* is amorphous, and may be white or coloured (yellow, red, pink, or purplish). It does not appear in the urine until this liquid is cooled, and instantly disappears on the application of heat.

*Urate of Soda* occurs in gout, and in the urine of persons labouring under fever who have been treated with carbonate of soda. It does not disappear so readily as urate of ammonia, on the application of heat. When examined by the microscope it is found to consist of mixed yellowish masses provided with projecting, generally curved, processes.

In discussing the pathological changes in the quantity of uric acid and urate of ammonia, Dr. Bird observes that—

"Excluding all abstract theories, whenever an excess of uric acid or its combinations with bases occurs in the urine, a normal quantity of water being present

(30 to 40 ounces in twenty-four hours), it may safely be inferred that one or other of the following states exist.

| | |
|---|---|
| A. Waste of tissue more rapid than the supply of nitrogenised nourishment, as in | Fever, acute inflammation, rheumatic inflammation, phthisis. |
| B. Supply of nitrogen in the food greater than is required for the reparation and supply of tissue, as in | Excessive indulgence in animal food, or the quantity of food remaining the same, with too little bodily exercise. |
| C. Supply of nitrogenised food not being in excess, but the digestive functions unable to assimilate it. | All the grades of dyspepsia. |
| D. The cutaneous outlet for nitrogenised excreta being obstructed, the kidney is called upon to compensate for this deficient function. | All or most stages of diseases attended with arrest of perspiration. |
| E. Congestions of the kidneys, produced by local causes. | Blows and strains of the loins, diseases of genital apparatus." |

Dr. Bird combats Liebig's hypothetical notions of the origin of an excess of uric acid.

"Professor Liebig recognises one great cause of the appearance of an excess of uric acid in the urine, founded on his theoretical views of the conversion of this substance into urea. It may be thus briefly enunciated, that as normally the insoluble uric acid first produced by the metamorphosis of tissues is, under the influence of oxygen conveyed in the red blood-discs, converted into soluble urea, whatever increases the number of blood-discs, or carriers of oxygen, or quickens the circulation, must cause the more complete conversion of uric acid into urea; and less of the former and more of the latter will appear in the urine. Conversely, whatever interferes with the perfection of oxygenation in the body, must necessarily produce an excess of uric acid. From this view, it follows that the quantity of uric acid ought to be positively or relatively to urea, decreased in

1. Fever.

2. Acute Phlegmasiæ.

3. Phthisis.

"And conversely it should be increased in

1. Chlorosis.
2. Anæmia.
3. Pulmonary emphysema.

"The only mode of testing hypotheses of this kind, emanating from a great and respected authority, is by clinical observation; and, so far as recorded facts are concerned, they fail altogether to give the slightest support to the ingenious theory of Professor Liebig.

"The labours of Edmund Becquerel in urinary pathology, furnish us with a mass of carefully recorded observations, which, made with no view of supporting or disputing any preconceived notions, are peculiarly entitled to respect. The numbers in the following table are calculated from some of the analyses alluded to, and point out the actual quantity of uric acid and urea excreted in the twenty-four hours, and the relative proportion they bear to each other, in several diseases.

| | Quantity in 24 hours of | | Ratio of uric acid to urea. |
|---|---|---|---|
| | Uric acid | Urea. | |
| | Grains. | Grains. | |
| Healthy urine (Becquerel's average).. .. | 8.1 | 255. | 1 : 31.48 |
| Chlorosis, minimum of five cases .. .. | 1.8 | 77.5 | 1 : 43. |
| Chlorosis, maximum of five cases .. .. | 6. | 172. | 1 : 29. |
| Pulmonary emphysema, extreme dyspnœa | 4.9 | 172. | 1 : 35.1 |
| Phthisis, tubercles softened .. .. .. | 9.1 | 66.7 | 1 : 7.33 |
| Phthisis, three days before death .. .. | 9.8 | 29.4 | 1 : 3. |
| Morbus cordis, with icterus .. .. .. | 9.82 | 73.3 | 1 : 7.6 |
| Acute hepatitis, with icterus .. .. .. | 11.18 | 61.6 | 1 : 5.6 |
| Icterus.. .. .. .. .. .. .. .. .. | 17.75 | 285.6 | 1 : 16.1 |
| Milk fever .. .. .. .. .. .. .. .. | 19. | 133. | 1 : 7.47 |

"From this table, we find that in chlorosis, a disease of anæmia, in which oxygenation of the blood, on the theory of Liebig, must be most imperfect; the uric acid, instead of being in excess, is positively and relatively below rather than above the healthy average. In pulmonary emphysema again, the same thing is observed, although, from the want of integrity in the function of respiration, uric acid ought to abound; whilst in acute hepatitis, and in phthisis, diseases in which, on Liebig's own showing, excessive oxygenisation is going on, the uric acid, both abstractedly and in relation to the urea, is at a minimum instead of a maximum. On this account, as well as for the reasons already alluded to, the theory of Liebig must, in the present state of knowledge, be deemed unsatisfactory." 81.

Of the medical treatment of the different forms of uric acid gravel, our author observes, that—

"Discarding altogether the existence of any specific agent for a disease which is rather symptomatic of another affection than really idiopathic, the therapeutical agents may be briefly referred to the following heads.

"1. *Attention to the function of the skin.*—The remarks already made on the effect of an arrest of perspiration furnishing a pabulum for the formation of a deposit, or by retaining in the circulation a substance capable of rendering uric acid insoluble, show the necessity of attending to this indication. I have repeatedly seen diaphoretics, warm clothing, the use of a flannel, and in winter, even a chamois-leather waistcoat, with friction by means of a flesh-brush or hair-glove, repeatedly remove a deposit of uric acid gravel, and in more than one instance, where even an hereditary taint existed from gouty or calculous progenitors." 90.

"My own experience induces me to regard the warm, or still better, the vapour bath, as the most valuable diaphoretic. The latter is readily employed in private practice by means of the very convenient and portable apparatus of M. Duval, which has for a long time superseded other forms of vapour-bath at Guy's Hospital. Actual diaphoresis is by no means necessary in the treatment of all cases of uric gravel; friction to the skin, and when persons are sufficiently robust, immersion in the cold-bath, followed by rubbing the surface of the body with a dry and rough towel, until reaction is produced, is often of great service.

"*Restoring the tone of the organs of digestion.*—By effecting this, a double object is attained; the perfection of the primary assimilation of the food by which the entrance of a crude nitrogenised matter, capable of being converted into uric acid, into the blood is prevented, and the prevention of the generation of any acid, the product of unhealthy digestion, which might be absorbed by the lacteals, and act as a precipitant of uric acid. This part of the treatment of calculous affections must be modified by the peculiarities of the case, and indeed is identical with that of the different forms of dyspepsia. Careful attention to the bowels, avoiding excessive purging, the use of minute doses of mercury, as of a grain of pil. hydrargyri or hydrarg. c. creta, with thrice that quantity of ext. conii, administered two or three times a day, with moderate doses of the carbonates of potassa or soda in the mist. gentianæ comp., if constipation exists, or in inf. calumbæ, or what is far better, from its action on the skin, inf. serpentariæ, will often effect immense relief. Where gastrodynia, with or without pyrosis, exists, the use of half a grain of argenti nitras, or one of argenti oxydum, immediately before a meal, will often check alike the gastric and renal symptoms. But the most important element in the treatment is a rigid attention to the quality and quantity of the ingesta, taking the utmost care to select those articles of diet which the patient can best digest, it being of far greater importance, in the majority of cases, to regard this, than to choose articles of food according to their chemical composition. A too bulky meal of animal or vegetable food is injurious to persons labouring under calculous dyspepsia, for whilst the former supplies too much nitrogen, both will become sources of mischief by overloading the digestive functions, and preventing the chylopoietic viscera doing their duty. In protracted cases, however, much good is derived by actually cutting off part of the supply of nitrogen. In this way I have seen a copious deposit of uric acid gravel disappear, after other measures had failed to give relief." 92.

"Moderate muscular exertion, and a due amount of exercise is quite essential in the treatment of this disease; for not only do they call into play some very important functions, but often improve the general health. Besides this, when the stomach is imperfectly able to digest nitrogenised food, exercise will often aid its assimilation by making a call upon the chylopoietic organ for supply for the want of tissue it produces.

"Among the remedies which appear most successful when the food is not converted into healthy chyle, and an unhealthy state of the blood from the presence of imperfectly assimilated matters results, the preparations of iron deserve notice. I have repeatedly seen copious deposits of uric acid in persons of low power completely disappear *pari passu* with the cure of the pseudo-chlorotic symptoms present, by the use of this important drug. The best mode of administering it, is in combination with a vegetable acid, as the stomach bears it well in this form, and it is probably more likely to enter the circulation. From six to twelve grains of the ammonio-citrate or ammonio-tartrate of iron taken thrice a day immediately after a meal in a glass of water, have been most successful. The solution of the sesqui-acetate of iron is also a very valuable preparation, but is often inconvenient to prescribe, in consequence of its not being of constant strength." 95.

Dr. Bird then proceeds to notice remedies which act as solvents of uric acid; and successively passes under review alkalies and their carbonates, biborate and phosphate of soda, benzoic and cinnamic acid.

"As the alkaline urates are far more soluble than the free acid, the employment of soda and potass with their carbonates has been long used in the treatment of uric gravel. They moreover exert a beneficial effect in neutralizing any free acid in the primæ viæ, and thus preventing a precipitant of uric acid reaching the kidneys. The liquor potassæ may be be employed in doses of half a drachm thrice a day: it is best taken about an hour after a meal, and may be conveniently administered in a little bitter ale, which conceals much of its disagreeable flavour, or in any bland vehicle. The carbonates of potass and soda are, however, far more agreeable, and perhaps more efficient remedies,—of these the bicarbonate of potass deserves the preference. It should be given thrice a a day in doses of ℈j. or ʒss. I think it appears to act best when taken in a glass of warm water. To make it more agreeable, I generally order, what I am accustomed to term to my patients, the artificial Vichy water, made by stirring ʒss. of bicarbonate of potass and gr. v. citric acid into a tumbler of lukewarm water. This mixture evolves enough carbonic acid to be 'sparkling,' and is generally taken with readiness.

"A very convenient mode of impregnating the urine with an alkali is to administer the potass or soda in combination with a vegetable acid, especially with the acetic, citric, or tartaric. The mode in which these act is easily explained; when acetate, citrate, or tartrate of potass are ignited, the acid absorbs oxygen, and is converted into carbonic acid and water, part of the former uniting with the alcali. In a similar manner are these salts decomposed during the process of healthy digestion; a carbonate finds its way into the circulation, and reaching the kidneys, renders the urine alcaline. If, however, the digestive powers are impaired, the vegetable acid is only partly decomposed, and in some few persons it escapes the influence of digestion altogether. 114 grains of tartrate of potass, 106 of citrate, and 99 of the acetate, absorb respectively 40, 48, and 64 grains of oxygen, to be converted into carbonate of potass and water. These salts may be administered by directing the use of the common saline powders made with carbonates of potass or soda and the citric or tartaric acid, in effervescence. When not contra-indicated, the use of strawberries, currants, and some other fruits containing alcaline citrates and malates, are capable of making the urine alcaline, and may be occasionally employed with advantage.

"Some persons cannot bear the use of free or carbonated alcalies without suffering severely in their general health, nor is their protracted use altogether without some ill effect. A flabby state of the muscles, and an anæmiated condition of the system, is frequently produced by the persistent use of alcaline remedies. Their injudicious employment may, as Dr. Prout has suggested, induce the formation of oxalic acid.

"Uric acid is soluble in a solution of borax, the biborate of soda,—more so, indeed, than in alcaline carbonates; and this salt may be taken for some time, at least by male patients, without producing any very injurious constitutional effects, and readily finds its way into the urine. On this account its administration has been suggested in cases of uric acid gravel, but it has not been much employed in this country. In women, this drug cannot be employed with impunity, as it certainly exerts a stimulant action on the uterus, and I have seen it in two instances produce abortion.

"The remarkable solvent action of phosphate of soda on uric acid, to which Liebig has lately directed attention, inspires a hope that its administration might be of use in cases of calculous disease, by impregnating the urine with an active solvent. All that is required to ensure this drug reaching the urine is to administer it in solution sufficiently diluted; ℈j. to ʒss. might be administered in any vehicle, as in broth or gruel, as when diluted, the phosphate tastes like common salt, and few persons object to its flavour. I have administered this drug in two very chronic cases of uric acid gravel, and in one with the effect of rapidly causing a disappearance of the deposit. This occurred in the person of a lady about forty years of age, who had, at my wish, for some weeks used the artificial Vichy water of the German Spa at Brighton without relief. The triple salt, ammonia-phosphate of soda, would perhaps be a more active remedy than the simple phosphate, but its disagreeable flavour constitutes one objection to its employment.

"Much attention has been lately drawn to the effects of benzoic acid in preventing the formation of uric acid, by the observations of Mr. Alexander Ure. When this acid or its salts are administered, they are acted upon by the stomach in a very different manner from the other vegetable acids. Instead of becoming oxidized, and being converted into carbonic acid, it combines with those nitrogenised elements which would otherwise have formed urea or uric acid, and is converted into hippuric acid. It has been stated that the quantity of uric acid falls, when the benzoic acid is administered, below the average quantity, or even disappears from the urine. This has been, however, shown by Dr. Garrod, to be an error, and that urea alone disappears. Be this as it may, it is certain that the acid does appropriate to itself some body rich in nitrogen to form hippuric acid; and experience has shown that, in cases where an excess of uric acid is secreted, the administration of this drug appears to limit it to about the normal quantity." 99.

Chap. IV. *Chemical Pathology of Uric Oxide.*—Uric oxide, called by its discoverer, Dr. Marcet, *xanthic oxide,* is a very rare kind of calculus, which, in nearly all the recorded cases, has occurred in children. Its formula is $C^5 N^2 H^2 O^2$. Concretions formed of it resemble those of uric acid, but their sections are characterised by a well-marked salmon or rather cinnamon tint. Uric oxide is distinguished from uric acid by its insolubility in a solution of carbonate of potash; by its dissolving in nitric acid with little or no effervescence; by its nitric solution having on evaporation a yellow residue; by its not being precipitated from its solution in strong sulphuric acid by the addition of water; and by some other less important characteristics. No crystalline arrangement of its parts can be detected by the microscope. The character of the urine depositing it, as well as the pathological and therapeutical indications derived from it, are entirely unknown; though Dr. Bird thinks that, from its remarkable similarity in composition to uric acid, the majority of the remarks made on the pathology of this acid might be applied to that of the oxide.

Chap. V. *Chemical Pathology of Purpurine.*—Purpurine, called by Simon *uro-erethrine,* is one of the colouring matters of the healthy urine. It must not be confounded with *murexid* (the purpurate of ammonia of Prout.) On account of its solubility in water, it never occurs as a deposit unless urate of ammonia be present, when this salt, in precipitating from urine, carries with it the great mass of purpurine, and in consequence acquires a more or less deep carmine tint. The deposit, which is amorphous, yields up its purpurine to alcohol. This would distinguish it from blood, with which it has sometimes been confounded. Moreover, alcohol is without action on murexid. The presence of purpurine in urine is best detected by adding a little hydrochloric acid to some of the urine previously warmed, a colour varying from lilac to purple, according to the quantity of colouring matter present, immediately occurs.

On evaporating urine containing purpurine to the consistence of an extract, and digesting it in alcohol, a fine purple tincture is obtained, the intensity of the tint being rather heightened by acids and diminished by alcalies.

Dr. Bird's observations on the pathological indications of purpurine are too valuable to be condensed, and we shall, therefore, give them entire.

" The presence of an excess of purpurine is almost invariably connected with some functional or organic mischief of the liver, spleen, or some other organ connected with the portal circulation. The appearance of a flesh-coloured deposit in the urine is the commonest accompaniment of even slight derangement of the hepatic function, as every case of dyspepsia occurring in gin-drinkers points out. The intensity of colour of the deposit appears to be nearly in relation with the magnitude of the existing disease. In the malignantly diseased, in the contracted, hobnail, or cirrhosed liver, the pink deposits are almost constantly present in the urine. They also are of frequent occurrence in the hypertrophy of the spleen following ague. The most beautifully coloured deposits I have seen have occurred in ascites connected with organic disease of the liver; and I think I have received some assistance in the diagnosis between dropsy depending upon hepatic and peritoneal disease, in the presence of the pink deposits in the former, and their general absence in the latter. I have occasionally seen the deposits in question occur in phthisis, when large quantities of pus were poured out from vomicæ, as well as in deep-seated suppuration, as in psoas abscess. But even in these cases, the portal circulation is probably more or less influenced. My experience, indeed, leads me to express a firm belief that an excess of purpurine is almost pathognomonic of disease in the organs in which portal blood circulates." 110.

CHAP. VI. *Chemical Pathology of Cystine.*—Cystine, termed by its discoverer, Dr. Wollaston, *cystic oxide,* is distinguished from all other urinary concretions by the sulphur it contains, and which amounts to about 26 per cent. The formula for cystine is $C^6\ H^6\ NO^4\ S^2$.

Calculi of cystic oxide are not laminated; but appear to form a mass confusedly crystallized throughout its substance. They are honey-yellow, semi-transparent, with a glistening lustre, somewhat like a confusedly crystallized mass of sugarcandy.

" Pure cystine is soluble in the mineral and insoluble in the vegetable acids; with the former it forms imperfect saline combinations, which generally leave by evaporation gummy masses or acicular crystals. It is readily soluble in ammonia and the fixed alcalies and their carbonates, but insoluble in carbonate of ammonia. Heated on platina-foil it burns, evolving a peculiar and disagreeable odour.

" A deposit of cystine may be distinguished from one of white urate of ammonia, by not disappearing on warming the urine, and from the earthy phosphates, by being insoluble in very dilute hydrochloric or strong acetic acid. The best character of cystine is its ready solubility in ammonia, mere agitation of some of the deposit with liquor ammoniæ being sufficient to dissolve it, and a few drops of the fluid, when allowed to evaporate spontaneously on a slip of glass, leaves six-sided tables of cystine. The ammoniacal solution, when kept for some time in a white glass bottle, stains it black, from the combination of the sulphur of the cystine with the lead in the glass.

" Another test has been proposed by Liebig, founded on the presence of sulphur; he directs the deposit suspected to contain cystine to be dissolved in an alcaline solution of lead, made by adding liquor potassæ to a weak solution of acetate of lead until the oxide at first thrown down is re-dissolved. On heating the mixture, a black precipitate of sulphuret of lead appears if cystine be present. All sulphuretted animal matters similarly treated yield black precipitates, and hence this test is useless, if any portions of albuminous or mucous substances are mixed with the deposit to be examined." 113.

Our author thus describes urine which deposits cystine.

" Most of the specimens of this variety of urine that I have met with, were pale yellow, presenting more of the honey-yellow than the usual amber tint of urine, not unfrequently possessing a somewhat oily appearance, like diabetic urine. The specific gravity of cystic urine is generally below the average, and is sometimes passed in larger quantity than natural. In one case (a child), in which Dr. Willis met with cystine, the urine was of a specific gravity of 1.030; but this is certainly unusual. It is often neutral, less frequently acid to litmus paper, but soon becomes alcaline by keeping.

"The odour of this form of urine is very peculiar, bearing in general a close resemblance to that of sweet-briar, and is sometimes rather powerful; less frequently the odour is fœtid, like putrid cabbage, owing, I suspect, to partial decomposition and evolution of sulphuretted hydrogen. In such specimens the colour has usually changed from pale yellow to green. In one case that occurred to me, the urine was actually of a bright apple green; it presented this tint for a few days, and the specimens subsequently voided were yellow." 114.

Cystine is very readily recognized by means of the microscope.

"When an ammoniacal solution of cystine is allowed to evaporate spontaneously on a piece of glass, it leaves crystals in the form of six-sided laminæ. These are probably exceedingly short hexagonal prisms. When the evaporation is slowly and carefully performed, these laminæ are transparent; but in general they are crystallised in a confused and irregular manner in the centre, the margins only being perfectly transparent. When examined by polarised light, these crystals, when sufficiently thin, present a beautiful series of tints, which are not observed when thick, on account of the high refracting power of cystine.

"When cystine occurs as a sediment, it is always crystallised, never under any circumstances being amorphous. Among the crystals, a few regular six-sided laminæ are often seen, but the great mass are composed of a large number of superposed plates, so that the compound crystals thus produced appear multangular, as if sharply crenate at the margin; and the whole surface is traversed by lines, which are really the edges of separate crystals. They thus resemble little white rosettes, when viewed by reflected light. These compound crystals always appear darker in the centre than at the circumference, which is sometimes quite transparent. Prisms of the triple phosphate are often mixed with the cystine, but on the addition of a few drops of acetic acid, they readily dissolve, leaving the rosettes of cystine unaffected." 117.

Dr. Bird agrees with Prout in referring cystine to an albuminous origin, and he observes that—

"Although but little is known of the pathological condition of the system which induces the formation of cystine, there is sufficient evidence before us to justify our expressing strong opinions of its essentially scrophulous and remarkably hereditary character. In one family alone, several members were nearly at the same time affected with cystine; and one instance exists in which it can be traced with tolerable certainty through three generations." 119.

In the treatment of cystine deposits the principal object to be attained is the improvement of the general health, and especially of the assimilative functions. To render the cystine, so long as it continues to be formed, soluble in the urine, nitro-hydrochloric acid has been recommended. Sea-bathing, exercise, nutritious and digestible diet, attention to the functions of the skin, and the use of iron, especially of the iodide, form Dr. Bird's principal measures for improving the general health.

Chap. VII. *Chemical Pathology of Oxalate of Lime* (*Oxaluria.*) Most of our readers are probably aware that to Dr. Bird is due the credit of having first shewn the comparative frequency of crystals of oxalate of lime in the urine; for, until the appearance of his researches on the subject, crystalline deposits of oxalate of lime were generally believed to be extremely rare. It is surprising how an error like this should have prevailed respecting a condition which Dr. Bird declares to be, with reference to this metropolis, of far more frequent occurrence than deposits of earthy phosphates. We feel indeed disposed to go a step beyond Dr. Bird, and to express our belief, founded on certain observations, that oxalate of lime deposits are not always to be regarded as evidence of morbid action.

The oxalic acid found in the urine can be derived from one or both of two sources only; viz. the food, or the metamorphoses of other bodies. Now we have reason to think that it is a much more frequent constituent of vegetable food than hitherto has been usually supposed; and being soluble, though indigestible, it readily enters the circulating mass, and is subsequently eliminated by the kidneys, in combination with a base. Curiously enough, Dr. Bird, in treating of the pathological origin of oxalate of lime, never once refers to the possible derivation of the acid of this salt from the aliment. He has drawn up a table to shew us how readily the supply of earthy phosphates can be derived from without, yet when treating of oxalate of lime—a salt which he admits to be of far more frequent occurrence—he totally omits to notice its probably similar origin. He tells us that oxalate of lime is always greatest in quantity after a full meal; that it is often absent in the *urina sanguinis*, or that passed on rising in the morning; and that it disappears under the influence of a carefully regulated diet, and re-appears on returning to the use of unwholesome food. Yet it never once seems to have occurred to him that, in many at least of these cases, the oxalic acid might be, and probably was, a ready-formed constituent of the alimentary substances employed by the patient!

Now we have satisfied ourselves that crystals of oxalate of lime may be readily obtained from the urine of persons in perfect health, if they have partaken either of sorrel sauce, or of tarts or puddings made of the leaf-stalks of rhubarb. A friend of ours in Paris, who has devoted much of his time to microscopic investigations under Gruby and Donné, tells us that when, for microscopical investigation, octohedral crystals of oxalate of lime were required, it was customary for himself and friends to dine off *fricandeau de veau à l'oseille;* and, in a few hours, an abundant supply of crystals was obtained. We mention this fact to shew with what facility oxalic acid may be obtained from healthy urine; and that we are not to assume, as Dr. Bird appears to do, that "certain definite ailments, all characterized by great nervous irritability," are necessarily connected with the occurrence of well-defined octohedral crystals of this salt in the urine.

We are indebted to the microscope for enabling us to discover the presence of oxalate of lime, not only in the urine, but also in the vegetable kingdom. It is well known that a distinguished English chemist submitted Russian rhubarb to a careful analysis, and failed to recognize the presence of oxalate of lime, although the microscope readily detects myriads of crystals of it; and an excellent microscopic observer (Mr. Edwin Quekett) has obtained from 100 grains of Russian rhubarb between 35 and 40 grains of this salt. If, then, oxalic acid be not frequently mentioned in the chemical analysis of esculent plants, it by no means follows that it is not a constituent of many of our vegetable foods; for, if present in small quantities, or only at certain seasons, it may readily escape detection by chemical agents. In such cases, the microscope may be most advantageously resorted to; for the purpose of detecting, if they be present, crystals of oxalate of lime. Now Schleiden, a most distinguished botanist and microscopical observer, tells us, that "oxalate of lime is most universally diffused. In no plant," he adds, "does it appear to be entirely wanting; and in many it is found in enormous quantities."—(*Grundzüge der wissenschaftlichen Botanik,* 2te Aufl., 1845.)

In order to comprehend the cause of the frequent, if not universal, presence of oxalic acid in plants, we must remind our readers that a vegetable is an apparatus of reduction, in which carbonic acid and water are decomposed and oxygen evolved; and that from carbonic acid is derived the carbon of the various proximate principles of plants; such as the acids, fecula, gum, sugar, oil, &c. Now, it is probable, as Liebig *(Annalen der Chemie,* Bd. XLVI.) has hinted, that the production of oxalic acid may be the first step in the reduction of carbonic acid, and in the formation of other vegetable principles. If we suppose that, by the presence of a base, and through the action of light, 12 equivalents of carbonic acid lose a fourth part of their oxygen, in consequence of the vital power acting on their elements, oxalic acid will be formed.

$$C_{12}\,O^{24} - O_6 = C_{12}\,O_{18} = 6 \text{ eq. of anhydrous oxalic acid.}$$

But oxalic acid does not exist in the anhydrous state. The hydrated acid contains one equivalent of water. Hence,

$$C_{12}\,O_{18} + H_6\,O_6 = C_{12}\,H^6\,O_{24} = 6 \text{ eq. of hydrated oxalic acid.}$$

Now if, by the continued action of the same influences, a further quantity of oxygen be abstracted, the oxalic acid is converted into tartaric or malic acid: tartaric acid, if only 9 eq. of oxygen are abstracted; malic acid, if 12 eq. are removed.

Hydrated oxalic acid, $C_{12}\,H_6\,O^{24} - O_9 = C_{12}\,H_6\,O_{15} = 3$ eq. tartaric acid.

„ „ „ $C_{12}\,H_6\,O_{24} - O_{12} = C_{12}\,H_6\,O_{12} = 3$ eq. malic acid.

By the mere abstraction of the elements of water from the elements of malic acid we obtain citric and lichenic acids.

Malic acid $C_{12}\,H_6\,O_{12} - HO = C_{12}\,H_5\,O_{11} = 3$ eq. citric acid.

„ „ $C_{12}\,H_6\,O_{12} - 3HO = C_{12}\,H_3\,O_9 = 3$ eq. lichenic acid.

We may regard tartaric, citric, and malic acids as combinations of oxalic acid with sugar, with gum, with woody fibre, or the elements of these.

Tartaric Acid. Oxalic Acid. Dry Grape Sugar.

$$2\,(C_{12}\,H_6\,O_{15}) = C_{12}\,O_{18} + C_{12}\,H_{12}\,O_{12}$$

By union with additional quantities of hydrogen (derived from the decomposition of water), any of these acids may serve for the formation of sugar, starch, gum, and lignin.

We have entered into these considerations for the purpose of shewing the probably extensive agency of oxalic acid in the chemical phenomena of vegetation; and also of accounting for the very general diffusion of this acid over the vegetable kingdom.

But while, on the one hand, we are anxious to show that oxalic acid is a constituent of most, if not of all plants,* and thereby to account for the by no means unfrequent occurrence of oxalate of lime in healthy urine, we beg to observe that there are many cases of disease in which the presence of sediments of oxalate of lime in the urine cannot be in this way accounted for; and in such we are obliged to admit that the acid has been generated in the system by some deranged condition of the assimilative process.

The formation of oxalic acid in the animal system is, however, due to changes of a reverse nature to those which originate this acid in plants. In the latter reducing, in the former oxidating, processes occur. In the vegetable, oxalic acid is formed by de-oxidation of carbonic acid: in the animal, it is produced by the oxidation of some carbonaceous compound.

"The ready conversion of uric into oxalic acid, under the influence of oxidizing agents, has been satisfactorily shown by Professors Liebig and Wohler; for when uric acid is heated with water and peroxide of lead, oxalic acid, carbonic acid, and allantoin, the peculiar ingredient of the allantoic fluid of the cow, are generated. The readiness with which, under certain circumstances, uric acid is converted into the oxalic, may be well illustrated by a fact which has been observed in connexion with the *guano* of South America, a substance now acquiring great celebrity as a manure. This contains, when recent, a considerable proportion of urate of ammonia, which salt, after a certain length of time, often during the voyage to this country, nearly wholly disappears, and is replaced by oxalate of ammonia." 139.

Dr. Bird gives the following directions for detecting the existence of oxalate of lime deposits:

"To examine urine for the purpose of detecting the existence of the salt under consideration, allow a portion passed a few hours after a meal to repose in a glass vessel. Decant the upper 6-7ths of the urine, pour a portion of the remainder into a watch-glass, and gently warm it over a lamp; in a few seconds the heat will have rendered the fluid specifically lighter, and induced the deposition of the crystals of oxalate, if any were present: this may be hastened by gently moving the glass, so as to give the fluid a rotatory motion, which will collect the oxalate at the bottom of the capsule. The application of warmth serves, also, to remove the obscurity arising from the presence of urate of ammonia, which is readily dissolved by exposing urine containing it, to a gentle heat. Having allowed the urine to repose for a minute or two, remove the greater portion of the fluid with a pipette, and replace it by distilled water. A white powder, often of a glistening appearance, will now become visible, and this, under a low magnifying power, as by placing the capsule under a microscope furnished with a half-inch object-glass, will be found to consist of crystals of oxalate of lime in beautifully-formed transparent octohedra, with sharply-defined edges and angles." 125.

"The crystals of the oxalate, when collected in the manner above directed in a watch-glass, are unaltered by boiling either in acetic acid or solution of potass. In nitric acid they readily dissolve without effervescence. The solution may be very readily watched under the microscope. When the oxalate is allowed to dry on a plate of glass, and then examined, each crystal presents a very curious appearance, resembling two concentric cubes, with their angles and sides opposed, the inner one transparent, and the outer black, so that each resembles a trans-

* Oxalate of lime constitutes nearly 50 per cent. of some lichens. *Variolaria faginea*, for example, contains 47·4 per cent. In such instances we may consider this salt as forming the skeleton of the plant.

lucent cube set in a black frame. This is best observed under a half-inch object-glass; as with a higher power this appearance is lost.

"In a very few cases the oxalate is met with in very remarkable crystals, shaped like dumb-bells, or rather like two kidneys with their concavities opposed, and sometimes so closely approximating as to appear circular, the surfaces being finely striated. These crystals are produced, in all probability, by a prolific arrangement of minute acicular crystals." 127.

With respect to the characters of urine containing oxalate of lime, our author observes that—

"In the great majority of cases, the urine was of a fine amber hue, often darker than in health, but never presenting to my view an approach to the greenish tint described by Dr. Prout as characteristic of the secretion during the presence of what he has described as the oxalic diathesis, unless red particles of blood were present. In a few cases the urine was paler than natural; and then was always of lower specific gravity. This, however, was in most instances but a transient alteration, depending upon accidental causes. In many instances a deposit of urate of ammonia, occasionally tinted pink by purpurine, fell during cooling. This I observed to be infinitely more frequent during the months of January to March than in the three succeeding months of this year: hence it in all probability depended upon the influence of cold upon the cutaneous functions, thus causing a large amount of azote, under the form of the urate, to be excreted by the kidney. The specific gravity of oxalic urine varies extremely; in rather more than half the specimens being, however, between 1·015 and 1·025. In eighty-five different specimens of which I have preserved notes, the ratio of the densities was as follows:—

| | | | |
|---|---|---|---|
| In 9 | specimens the specific gravity ranged from | | 1.009 : 1.015 |
| In 27 | ditto | ditto | 1.016 : 1.020 |
| In 23 | ditto | ditto | 1-021 : 1-025 |
| In 26 | ditto | ditto | 1.025 : 1.030." |

"Many of the specimens of oxalic urine gave a precipitate with salts of lime, insoluble in acetic acid, and consisting of oxalate of lime. This, in some instances at least, depended on the presence of oxalate of ammonia, and delicate acicular crystals of this salt occasionally formed upon the edge of the capsule by spontaneous evaporation.

"The acidity of these specimens was always well marked, often far more so than in health, and never being absent. I have not yet met with a single case in which an alcaline, or even positively neutral, state existed.

"A greater increase in the quantity of urea, than the density of the urine would have led us to suspect, was frequently found; indeed, I have scarcely met with a specimen in which, when the density was above 1.015, distinct indications of an excess of urea were not met with. In twenty-four of the eighty-five specimens above referred to, so large a quantity was present, that very rapid, and in some almost immediate, crystallisation ensued on the addition of nitric acid. In general, in cases where the greatest excess of urea was present, the largest and most abundant crystals of the oxalate were detected." 131.

The following table shows the proportion of cases in which oxalate of lime was found complicated with other deposits.

"Out of the eighty-five cases before referred to

| | | |
|---|---|---|
| Oxalate was present unmixed in | 43 | cases. |
| Mixed with urate ammonia in | 15 | ,, |
| Mixed with uric acid | 15 | ,, |
| Mixed with triple phosphate | 4 | ,, |
| Phosphate deposited by heat | 8 | ,, |
| | 85 | |

"In one of the specimens containing the triple phosphate, the application of heat produced a deposit of the earthy salts.

"One very constant phenomenon was observed in the microscopic examination of oxalic urine, viz. the presence of a very large quantity of epithelial scales; it was, indeed, the exception to the general rule to meet with this form of urine free from such an admixture. So constantly was it found, that repeatedly a white deposit of epithelium has often attracted my attention, and led to the suspicion of the probable presence of oxalate of lime." 132.

In no case has Dr. Bird detected sugar in urine containing oxalate of lime; and, therefore, the popular notion of the connection of this deposit with saccharine matter is unsupported by any evidence.

On the therapeutical indications for the treatment of oxaluria, Dr. Bird makes the following observations.

"The treatment, in the majority of cases, is very successful; a few only resisting all the plans which were adopted. As a general rule, the functions of the body, where obviously imperfect, should be corrected, the general health attended to by the removal of all unnaturally exciting or depressing influences, the skin should be protected from sudden alternations of temperature by a flannel or woollen covering, and the diet carefully regulated. This has generally consisted of well-cooked digestible food, obtained in about equal proportions from the animal and vegetable kingdom; all things which tend to produce flatulence being carefully avoided. The drink should consist of water, or some bland fluid, beer and wine being excluded, especially the former, unless the patient's depression render such positively necessary. A very small quantity of brandy in a glass of water has generally appeared to be the most congenial beverage at the meals. The administration of nitric acid, as suggested by Dr. Prout; or what appeared to be preferable, the nitro-hydrochloric acid, in small doses, in some bitter infusion; or, laxative mixture, as the mistura gentianæ comp., was, with minute doses of mercury, generally successful, if continued a sufficient length of time. In cases where these failed, active tonics, especially the sulphate of zinc, and where the patient was anæmiated or chlorotic, the salts of iron in very large doses, appeared to be of great use, by subduing the irritable state of the nervous system. The shower-bath, by acting in a similar manner, has been also of great service. There is one remedy which appears to exercise a marked influence over the characters of the urine, and which, from the small amount of experience I have had with it, seems to hold out the probability of its great utility in the disease under consideration: I allude to the colchicum, which, it is now generally admitted, exerts an immense influence over the organic system of nerves, and the functions under its control. The character of the urine is remarkably influenced by this drug, an excess of uric acid generally being present during its administration; and in two cases, in which oxalate of lime existed in abundance before its employment, uric acid appeared after a few days as a deposit, and nearly entirely replaced the oxalate; a circumstance generally observed during the successful treatment of this disease by other remedies. In no case have I seen the disease suddenly yield; it has generally slowly disappeared *pari passu* with the decrease in number and size of the crystals of the oxalate." 144.

If the views we have advocated be correct, a larger amount of animal food is indicated than Dr. Bird recommends.

CHAP. VIII. *Chemical Pathology of the Earthy Salts.*—Under this head Dr. Bird examines deposits composed of phosphate of lime, ammonio-phosphate of magnesia, carbonate of lime, and silicic acid. The two latter substances are comparatively unimportant. Carbonate of lime sometimes occurs in small quantities in deposits of earthy phosphates when the urine is alkaline, and probably owes its origin to the decomposition of the earthy phosphate by carbonate of ammonia. Silicic acid is so rare that many writers doubt its existence in the urine.

Deposits of the earthy phosphates are always white, unless coloured with blood. They are soluble in hydrochloric acid and are insoluble in ammonia or liquor potassæ. Nor are they soluble in the urine by heat. Phosphate of lime occurs as an opake amorphous powder. The triple phosphate (phosphate of magnesia and ammonia) forms a crystalline deposit called *white gravel.* The neutral triple phosphate (HO, $NH^4$ O, MgO, $P^2$ $O^5$) occurs in prisms, stellæ, and penniform crystals. The prisms are well defined with sharp and well defined angles and edges. The basic triple phosphate ($NH^4O$, 2 MgO, $P^2$ $O^5$) occurs in stellar and foliaceous crystals.

With respect to the pathological indications of the phosphates, Dr. Bird justly observes that—

"The occurrence of deposits of the earthy phosphates in the urine, must be regarded as of serious importance, always indicating the existence of important functional, and too frequently, even of organic mischief. One general law appears to govern the pathological development of these deposits, viz. that they always exist simultaneously with a depressed state of nervous energy, often general, rarely more local, in its seat. Of the former, the result of wear and tear of body and mind in old people, and of the latter the effects of local injury to the spine, will serve as examples. It is true, that in the majority of these cases there is much irritability present, there is often an excited pulse, a tongue white on the surface and red at the margin and tip, with a dry, often imperspirable, occasionally hot skin. Still it is irritability with depression, a kind of erythism of the nervous system, if the expression be permitted, like that observed after considerable losses of blood." 176.

Our author recognises at least four different pathological conditions connected with deposits of the earthy phosphates.

"A. Cases in which dyspepsia, with some febrile and nervous irritation, exists independently of any evidence of antecedent injury to the spine.

"B. Cases characterised by high nervous irritability, with a varying amount of marasmus, following a blow or other violence inflicted on the spine, but without paralysis.

"C. Cases in which the phosphatic urine co-exists with paraplegia, the results of spinal lesion.

"D. Cases of diseased mucous membrane of the bladder." 192.

The treatment of the first class of cases must be rather directed by general principles than limited to the solution of the phosphatic deposits.

"It is true that by the persistent administration of acids the deposit may disappear for a time, but the ailment goes on; all that is effected by such treatment is to mask a symptom, and an important one, of the progress of the malady. After having attended to the morale of the case, as far as possible rousing the patient from any morbid influence excited in his mind, whether real or imaginary; the next thing is to attend to the general health. The bowels should be freed from any unhealthy accumulation by a mild mercurial laxative, as a few grains of pil. hydrarg., followed by a dose of rhubarb or castor-oil; but all active purging should be avoided, as it generally aggravates the distress of the patient, and decidedly interferes with the success of the treatment. A combination of a tonic-laxative with a sedative may then be administered, as tinct. hyoscyami et sp. ammon. aromatici āā ♏xx—ʒss. ex mist. gentianæ co. ℥j. ter in die. If the bowels be irritable, the inf. cascarillæ, or inf. serpentariæ, may be substituted for the mist. gentianæ comp. Should gastrodynia exist, great relief will be obtained by the administration of half a grain of oxyde of silver, made into a pill with confection of opium, before a meal. The diet should be very carefully regulated, all bland nutritious articles of food being preferred; vegetables should be avoided, and in general a small quantity of good sherry may be allowed. By a plan of treatment of this kind, the patients generally do well, and the phosphates and excess of urea vanish from the urine. As the patient approaches convalescence, much good is often effected by the use of sulphate of zinc in gradually increasing doses, beginning with a grain thrice a day, made into a pill with a little ext. hyoscyami, or ext. gentianæ, and increasing the quantity every three or four days, until five grains or more are taken at a dose. Under the use of the zinc, I have seen many cases do well, whose symptoms approached in severity and character those of mild delirium tremens. I need hardly say that change of scene and occupation are important adjuvants to our medical treatment." 194.

The second class of cases are far less amenable to treatment.

"In these, the phosphatic deposit is often copious and sometimes consists nearly exclusively of phosphate of lime; the lumbar pain and weight are considerable, the skin often dry and scarcely perspirable; in some cases, indeed, I have seen it look as if varnished; the tongue sometimes white, is often red; the thirst often great; indeed, the general appearance of the case closely resembles one of diabetes. The urine is generally more copious than natural, frequently pale, and of a specific gravity below the average. On investigating the patient's history, some evidence of a previous strain or wrench of the back, or a blow over the spine, is always elicited. These patients are seldom hypochondriacal; but intense irritability of temper, and a painfully anxious expression of countenance and manner, are almost invariably present.

"In the treatment of these cases, the great end and aim must be to subdue the morbidly irritable state of the brain and nervous system; and subsequently, by a generous diet and persistent use of those tonics which appear especially to exert their influence on the organic nerves, as silver, bismuth, zinc, &c., to endeavour to restore the assimilative functions to their due vigour. Besides the general indications to be fulfilled by regulated diet, amusements, exercise, &c. the use of narcotics, especially of opium, or the preparations of morphia, should be regarded as of the highest value; and we are indebted to Dr. Prout for first directing the attention of the profession to their use." 199.

"Cases occasionally occur in which the symptoms are of a much milder character, but which insidiously go on to the formation of a calculus. It is in these in particular that the use of acids is called for, to hold the phosphatic salts in solution, and prevent their being moulded into a concretion in the pelvis of a kidney. Unfortunately there is a great uncertainty attending their use; sometimes the mineral acids appear to reach the urine and destroy its alcaline character; often, however, even their continued employment appears to be utterly ineffectual in rendering the urine acid. So far as I have watched cases of this kind, the nitric acid has appeared to produce the smallest amount of gastric derangement, and to render the urine acid, or at least diminish its alcaline reaction. In one case lately, in which the nitric acid could not be borne, the phosphoric appeared to succeed." 201.

In the third class of cases, the deposition of the phosphates is a sure symptom of a grave and serious lesion, and must be treated according to the particular disease existing.

The fourth class of cases, or "those in which the phosphates are probably entirely secreted with unhealthy mucus by a diseased lining membrane of the bladder," are familiar to every practitioner.

"One point of great practical consequence must be borne in mind in forming a prognosis from the state of the urine, viz. not to regard it as ammoniacal, because the odour is offensive; and not to consider the deposit as purulent because it looks so. A piece of litmus paper will often show the urine to be really acid, and microscopic inspection often proves that the puriform appearance of the urine is owing to abundance of phosphates with mucus. For want of these precautions I have seen one or two cases regarded as almost hopeless, which afterwards yielded to judicious treatment. It is quite certain that the mucous membrane of the bladder may, under the influence of chronic inflammation, secrete so much of the earthy phosphates and unhealthy mucus as to render the urine puriform and offensive without having necessarily undergone any structural change.

"A few cases have occurred to me in practice, in which the kind of urine just referred to was secreted for a long time, and yet yielded readily to treatment. In these, the greatest good has arisen from freeing the bladder from the phosphates which appear almost to incrust it, by acid injections. In this way cases have occasionally yielded which have quite defied all other treatment." 207.

In one case which Dr. Bird relates, great benefit was obtained by the daily careful injection of acidi hydrochlorici ♏x, with vini opii ♏xx. in barley-water.

Chap. IX. *Deposits of Black or Blue Colouring Matter.*—Exclusive of purpurine, and the colouring matters of the bile and blood, certain other pigmentary deposits, products of morbid action, are occasionally met with in the urine. Five of these are noticed by Dr. Bird; three of them are blue, and two are black. Of these the composition (which we subjoin) of two only has been ascertained.

| | |
|---|---|
| Blue deposits.... | Cyanorine. |
| | Indigo $C^{16}\ NH^{5}\ O^{2}$ |
| | Prussian Blue $Fe^{7}\ C^{18}\ N^{9}$ or $Fe^{7}\ Cy^{9}$ |
| Black deposits .. | Melanourine. |
| | Melanic Acid. |

a. *Blue Deposits.*—Blue urine was occasionally met with and noticed by the ancients; but its colouring principles have only been examined of late years.

*Cyanourine,* discovered by Braconnot, is a dark blue powder, which combines with acids, forming a solution which, when the acid is at the minimum is brown, when at a maximum, is of a magnificent carmine red. *Indigo* forms a deposit in the urine of epileptics who have been taking this colouring matter remedially. In some cases perhaps it may be generated in the system. It is soluble in oil of vitriol, and is bleached by chloride of soda. *Prussian blue* (called by Dr. Bird, who on this point adopts the erroneous nomenclature of the London Pharmacopœia, *percyanide of iron*) is sometimes artificially produced in the urine by giving to a patient who has been taking some preparations of iron, a few doses of ferrocyanide of potassium (yellow prussiate of potash). Occasionally it is also met in the urine, in other cases, generally where iron has been employed. Liquor potassæ destroys the blue colour of Prussian blue, and yields a yellow solution which gives a blue precipitate with the sesqui-salts of iron, and reddish brown with the sulphate of copper. The pathological and therapeutical indications of the blue pigments of the urine are quite unknown.

We take this opportunity of stating that, we have reason to believe that the bluish green film, which is often seen covering the purulent secretion of indolent ulcers, and which stains the dressings green, contains Prussian blue.

B. *Black Deposits.*—Very little is known of the black deposits (*melanourine* and *melanic acid*) of the urine. We feel assured that, in some of those cases in which patients have been said to pass black urine, imposition has been practised on the medical attendant. One case occurred to ourselves. We had some black urine brought to us for examination by a medical friend, who told us it was evacuated by a young lady, and that the same was invariably passed after the patient had been cupped! On examination, we found the colouring matter to be ink; and the young lady ultimately confessed that, to deter her medical attendants from a repetition of the cupping, she had, on several occasions, emptied the ink-bottle into the chamber utensil.

In those cases in which black urine has been really secreted by the kidneys, it is probable that the pigment was some modification of the colouring matter of the blood. Nothing whatever is known of the pathological and therapeutical indications of black urine.

CHAP X. *Noncrystalline Organic Deposits.*—In this chapter Dr. Bird passes successively under review the following substances which occur in the urine: 1, the elements of blood; 2, purulent deposits; 3, mucus; 4, organic globules; 5, epithelium; 6, milk; 7, fatty matter; 8, spermatozoa; 9, torulæ; 10, vibriones. In the detection of these deposits microscopical examination is indispensable.

1. *Elements of Blood.*—Serum of the blood may occur in the urine either alone, as in Bright's disease and in the anasarca which results from scarlatina,—or it may be accompanied by the red particles, as in cases of fungus hæmatodes of the kidneys. When the quantity of blood effused is large, it coagulates and forms masses like pieces of currant-jelly. Urine containing albumen becomes opaque by heat, as well as on the addition of nitric acid. The deposit stained by heat should not disappear on the addition of a few drops of nitric acid. This distinguishes albumen from the earthy deposits sometimes obtained by boiling urine. Pariset's test for the detection of hæmatosine is the following: "Boil the urine and filter it. Brown coagula of hæmatosine and albumen will be left on the filter; pour on these liquor potassæ, and if hæmatosine be present a greenish solution will pass through, from which hydrochloric acid will precipitate white coagula of protein."

After describing the microscopical characters of the blood-corpuscles, the author proceeds to notice the pathological and therapeutical indications of blood, or its elements in the urine. Our space prevents us from giving more than the following extract from this portion of our author's work.

"No remedy has, however, appeared to me to be of such extraordinary value in the treatment of hæmaturia as gallic acid. I have seen this drug arrest for many weeks bleeding from an enlarged (and fungoid?) kidney, after all other remedies had failed. It should be given in doses of five grains in a draught, with mucilage, and a little tinct. hyoscyami, and repeated at short intervals. This drug really acts as a direct astringent, reaching the capillaries of the kidney, and finding its way into the urine, which soon becomes so impregnated with it, as to be changed into ink on the addition of a few drops of tinctura ferri sesqui-chloridum." 234.

2. *Purulent Deposits.*—The occurrence of pus in the urine indicates the existence of suppurative inflammation in some parts of the urinary apparatus, and the treatment will of course depend upon the nature of the disease. The pus-globule is a nucleated cell larger than a blood-disk, and floats in an albuminous liquor (*liquor puris*) not spontaneously coagulable.

3. *Mucus.*—Mucous particles are not distinguishable from the pus-globule, with which probably they are identical.

"Where mucus and pus essentially differ is not in the nature of the particles, but in the fluid secreted with them, and in which they float; the *liquor puris* being albuminous and coagulable by heat, the *liquor muci* not being affected by it." 242.

Mucous deposits in general indicate irritation or inflammation of the genito-urinary mucous membrane; and the treatment must depend on the nature of the exciting cause.

3. *Organic Globules.*—Dr. Bird describes two of these. The *large* organic globule resembles the pus or mucous globule, but is unaccompanied with the characteristic albuminous fluid of pus, and with the glairy fluid of mucus. It has been found in every case of ardor urinæ. The *small* organic globule contains no traces of nucleus or traces of granulation. It is very rare. Dr. Bird found it in the urine of two women during menstruation.

4. *Epithelium.*—Epithelium-cells occur abundantly when deposits of oxalate of lime exist in the urine. They are regularly oval or irregularly angular flattened cells, and contain a well-marked central nucleus.

5. *Milk.*—Dr. Bird remarks that no satisfactory case is recorded, by any observer of credit, in which milk has been discovered in the urine. All the cases of milk-like urine, where no fraud has existed, are instances of phosphatic, purulent, or fatty urine. The pellicle which is found on the urine of pregnant, and probably also of suckling women, and which has been called *kiestein,* consists, according to Dr. Bird, of fat, (butter?), numerous crystals of triple phosphate, and an animal matter allied to, or identical with, casein, and which Dr. Stark has proposed to call *gravedine.*

6. *Fatty Matter.*—All the genuine specimens of fatty urine that have occurred to Dr. Bird have been opake, like diluted milk, and, in the majority of instances, have spontaneously gelatinised like so much blanc-mange, on cooling. To these the term *chylous urine* has been applied by Dr. Prout. By agitating the fresh urine with an equal bulk of ether in a tube the fat is dissolved, and by repose a yellow ethereal solution of it will float on the top of the urine, which, by thus losing the fat, becomes nearly transparent. If the solution be decanted, and suffered to evaporate spontaneously in a watch-glass, a solid butter-like fat, having a rancid odour, is obtained. The urine also contains albumen [fibrin ?] in a spontaneously coagulable form. It occurs in patients who are disposed to obesity.

7. *Spermatozoa.*—If any of the spermatic secretion be present in urine it subsides, by repose, to the bottom of the vessel, and may be mistaken for mucus ; but the microscope detects in it spermatozoa. Their presence of course proves that the seminal secretion must have been mixed with the urine, and often enables the physician to detect a source of exhaustion previously concealed from him.

8. *Torulæ.*—When saccharine urine is undergoing the vinous fermentation, very minute nucleated cells of an oval form are developed in it. After some time articulated filaments make their appearance. All these different bodies Dr. Bird includes under the denomination of *Torulæ*, though it is obvious enough to those who are familiar with these various structures, that they are not all referable to one genus ; and in the figures which Dr. Bird has published, we detect two, if not three different species.

Dr. Bird observes that—

"The most trustworthy tests for the detection of sugar in urine depend for their action upon the reducing action of sugar on salts of copper, or upon the decomposition of the sugar by alcalies.

"1. *Trommer's test.*—Add to the suspected urine in a large test-tube just enough of a solution of sulphate of copper, to communicate a faint blue tint. A slight deposit of phosphate of copper generally falls. Liquor potassæ must then be added in great excess; a precipitate of hydrated oxide of copper first falls, which re-dissolves in the excess of alcali, if sugar be present; forming a blue solution like ammoniuret of copper. On gently heating the mixture to ebullition, a deposit of red suboxide of copper falls if sugar be present.

"2. *Capezzuoli's test.*—Add a few grains of blue hydrated oxide of copper to urine contained in a conical glass vessel, and render the whole alcaline by the addition of liquor potassæ. If sugar be present, the fluid assumes a reddish colour, and in a few hours the edge of the deposit of oxide assumes a yellow colour which gradually extends through the mass, from the reduction of the oxide to a metallic state (suboxide ?).

"3. *Moore's test.*—This very easily applied test was lately proposed by Mr. Moore, of the Queen's Hospital, Birmingham, and depends for its action on the conversion of colourless diabetic (grape) sugar into brown melassic (or perhaps sacchulmic) acid under the influence of a caustic alcali. Place in a test-tube about two drams of the suspected urine, and add nearly half its bulk of liquor potassæ. Heat the whole over a spirit-lamp, and allow actual ebullition to continue for a minute or two; the previously pale urine will become of an orange-brown, or even bistre-tint, according to the proportion of sugar present. This test appears to be remarkably free from sources of fallacy, as boiling with liquor potassæ rather tends to bleach non-saccharine urine than to deepen its colour." 279.

Trommer states that, by his test, grape sugar may be readily distinguished from cane sugar; and Dr. Ure (*Pharmaceutical Journal,* vol. ii. p. 12) has proposed it as a means of detecting the fraudulent adulteration of Muscovado sugar with potato-sugar. We have, however, found that it is quite inapplicable for this purpose; since the uncrystallizable cane sugar (treacle) contained in genuine Muscovado gives the same results as starch-sugar. Having some reason to suspect the applicability of Trommer's test to the detection of sugar in the urine, we resolved to subject Dr. Bird's "most trustworthy test" to careful examination.

For this purpose we provided ourselves with some urine which we believed to be healthy, and cautiously added to it, first a solution of sulphate of copper, and then excess of caustic potash, as directed by Dr. Bird. Instead of obtaining a precipitate of the hydrated oxide of copper, as his statement leads us to expect, we got "a blue solution like ammoniuret of copper," which, according to Dr. Bird, indicated the presence of sugar. The mixture was then heated to ebullition, when the blue colour disappeared, and a reddish deposit was formed, thus farther proving, according to Dr. Bird, the presence of sugar.

But as we had more confidence in the healthy quality of the urine under examination than in this "most trustworthy test," we made another experiment in elucidation of the preceding one. We dissolved some urate of ammonia (fæces of the boa constrictor) in warm water, and treated the solution as we had previously treated the urine: the same results were obtained. On the addition of excess of caustic potash, the urate of ammonia was decomposed; and the liberated ammonia combining with the oxide of copper formed a blue solution of ammoniuret of copper; and by ebullition, we obtained a red cupreous precipitate. We then collected some urate of ammonia from urine, dried it, dissolved it in water, and then subjected the solution to Trommer's test; the result was as before. We have, therefore, no hesitation in cautioning our readers against pinning their faith on Trommer's test for diabetic sugar, albeit Dr. Bird does call it a "most trustworthy test."

9. *Vibriones.*—Dr. Bird states that minute animalcules of the genus Vibrio (V. Lineola ?) are sometimes developed in the urine, so soon after passing as to lead to the idea that their germs must have existed in the urine whilst in the bladder. They have been found in abundance in cases of syphilitic cachexia and in mesenteric diseases.

Chap. XI. *Therapeutical Employment of Remedies influencing the Kidneys.*—In the eleventh and last chapter of his work, Dr. Bird examines the assumed capricious influence of diuretics, and lays down three "general laws" which regulate the action of these remedies.

"Law 1st. All therapeutical agents intended to reach the kidneys must either be in solution when administered, or capable of being dissolved in the fluids contained in the stomach or small intestines, after being swallowed.

"Law 2nd. Bodies intended to reach the kidneys must, to ensure their absorption, have their solutions so diluted as to be of considerably lower density than either the liquor sanguinis, or serum of blood.

"Law 3. If a sufficient quantity of water cannot be received into the small intestines, or the circuit through the portal system in the vena-cava ascendens, or thence through the lungs and heart into the systemic circulation, be obstructed, or if there be extensive disorganisation of the kidneys, the due secretions of urine cannot be effected." 290.

To the enunciation of the first of these propositions, or "laws," as Dr. Bird calls them, no one can object; since the facts which it embraces have long been known and practically acted on. The second "law," however, is of a more hypothetical nature. It was first propounded by Liebig, and is founded on observation of the phenomena included under the denomination of endosmosis and exosmosis. When a piece of dead animal membrane is interposed between two liquids of unequal density, two opposite but unequal currents are established through the membrane; the stronger one in general flows from the lighter to the denser fluid; the weaker one, from the denser to the lighter fluid. The same phenomena are assumed to take place with living animal membrane; and, therefore, when saline substances are intended to act on the kidneys, they are recommended to be given in the form of solution, the density of which must be less than that of the serum of the blood; otherwise, instead of being absorbed, the solution will abstract liquid from the coats of the alimentary canal, and act as a hydragogue purgative. This application of the facts of endosmosis to the explanation of the diuretic and hydragogue action of saline substances is exceedingly ingenious and plausible; but must be received with considerable caution; as indeed should all physical and chemical explanations of the phenomena of living beings. It reduces the alimentary canal to the condition of a dead animal membrane; and supposes that the serum of the blood escapes through the parietes of the vessels; for it is certain that when two dissimilar fluids are separated by a dead membrane, double permeation is effected. Moreover, it overlooks one important fact, established by Dutrochet, namely, that in the case of certain liquids the stronger current is from the denser towards the lighter fluid.

Dr. Bird's third law is a proposition made by Dr. Barlow in the *Guy's Hospital Reports*, for October, 1844.

We shall conclude our observations on this chapter of Dr. Bird's work, by quoting his practical conclusions drawn from the laws above stated.

"1. Whenever it is desirable to impregnate the urine with a salt, or to excite diuresis by a saline combination, it must be exhibited in solution, so diluted as to contain less than five per cent. of the remedy, or not more than about twenty-five grains in an ordinary draught. The absorption of the drug into the capillaries will be ensured by a copious draught of water, or any diluent, immediately after each dose.

"2. When the urine contains purpurine, or other evidence of portal obstruction exist, the diuretics or other remedies employed should be preceded or accompanied by the administration of mild mercurials,—taraxacum, hydrochlorate of ammonia, or other cholitic remedies. By these means, or by local depletion, the portal vessels will be unloaded, and a free passage obtained to the general circulation.

"3. In cases of valvular or other obstructions existing in the heart and large vessels, it is next to useless to endeavour to excite diuretic action, or appeal to the kidneys by remedies intended to be excreted by them. The best diuretics here will be found in whatever tends to diminish the congested state of the vascular system, and to moderate the action of the heart; as digitalis, colchicum, and other sedatives, with mild mercurials." 293.

In concluding our notice of Dr. Bird's book, our readers will expect from us a formal opinion of its merits and demerits. That we consider it an important work will be evident from the great space which we have devoted to its critical analysis. But it is disfigured by several blemishes. It appears to us to have been got up in a very hasty manner, and without time being allowed for the reading of the proof-sheets. At least in no other way can we account for the numerous typographical and other errors which everywhere meet the eye. Our author is as unfortunate in spelling names and quoting books as the French writers proverbially are. With some words he seems to be undecided as to the proper etymology. Thus we have "octohedron" in one line, and "octahedron" in the next one. The French and German words are regularly murdered by him. He spells French words in German fashion (ex. gr. "*chimie*" he writes "*chemie*"), while German words are spelled in French fashion (ex. gr. "*de*" for "*der*.") In the proper employment of capital and small letters gross errors are constantly committed. Thus in quoting the titles of German books, capital letters are frequently employed when they are inadmissible; and in quoting botanical names, the same errors are committed (ex. gr. *Pisum Sativum* is put for *Pisum sativum; Phaseolus Vulgaris* for *Phaseolus vulgaris*.) The latter error is constantly made by persons unacquainted with the rules followed by botanical writers; but Dr. Bird, as a lecturer on Materia Medica, deserves reproof for this fault. At p. 146 a very serious error occurs, "℥" being substituted for "ʒ." *One ounce of strong nitric acid, and one ounce of strong muriatic acid* being ordered instead, we presume, of *one drachm* of each of these acids. We trust that in another edition of the work (which we doubt not will be called for) these and numerous other errors will be rectified.

Notwithstanding the blemishes which we have considered it our duty to notice, we conscientiously believe it to be a very useful work. It contains an excellent summary of what is known on the subject of which it treats; and we advise all our readers, who desire to be up to the present state of the chemical pathology of the kidney, and who wish to avail themselves of every practical novelty, to purchase it. It presents us with a clear, concise, and judicious exposition of the entire subject of urinary deposits, and should be in the library of every intelligent practitioner.

